# Adolescents' perceptions and user experiences with a virtual reality-based alcohol prevention tool in Germany: A focus group study

**DOI:** 10.3389/fpubh.2023.1054015

**Published:** 2023-03-10

**Authors:** Robert Hrynyschyn, Christina Prediger, Patricia Lyk, Gunver Majgaard, Stefanie Maria Helmer, Christiane Stock

**Affiliations:** ^1^Charité – Universitätsmedizin Berlin, corporate member of Freie Universität Berlin and Humboldt Universität zu Berlin, Institute of Health and Nursing Science, Berlin, Germany; ^2^Leibniz Science Campus Digital Public Health, Bremen, Germany; ^3^University of Southern Denmark, Game Development and Learning Technology, The Maersk Mc-Kinney Moller Institute, Odense, Denmark; ^4^University of Bremen, Human and Health Sciences, Bremen, Germany; ^5^University of Southern Denmark, Unit for Health Promotion Research, Esbjerg, Denmark

**Keywords:** virtual reality, user experience, alcohol prevention, adolescents, focus group, qualitative research

## Abstract

**Background:**

Excessive alcohol consumption is a major public health problem, with substance use early in life contributing to higher levels of use later in life. Virtual reality (VR) is an innovative technology for alcohol prevention among adolescents that could solve the problem of insufficient outreach to the target group of young people. The co-created German *Virtual LimitLab* simulation is one of the few examples of VR-based alcohol prevention tools and consists of a virtual house party simulation. The aims of *Virtual LimitLab* are to increase the users' awareness of how social pressure can influence their own decision-making as well as to enable various actions and communication strategies in order to train competencies when dealing with alcohol. The present study thus aims to explore adolescents' content- and technique-specific perceptions of *Virtual LimitLab* in order to gain insights into user experiences and to test the prototype with the German target group.

**Methods:**

Four semi-structured focus groups with adolescents aged 15–18 years (*n* = 13) were conducted and analyzed using thematic analyses. A user experience questionnaire (UEQ–S) was applied in order to quantitatively assess adolescents' satisfaction with *Virtual LimitLab*.

**Results:**

Three main themes were identified (*VR experience, content*, and *technical aspects*). Participants positively assessed both the content and the technical aspects of *Virtual LimitLab*. This trend was also seen by the UEQ–S data, which yielded positive ratings for both pragmatic and hedonic quality. The broad variety of options in the simulation that allow the user to try new behaviors was perceived particularly positively. In general, *Virtual LimitLab* was regarded as an innovative tool that encourages adolescents to think critically about their personal alcohol consumption. Technical errors in the simulation and users' difficulties in identifying with the simulation were the main points of criticism.

**Conclusions:**

Feedback from adolescent users revealed positive and therefore promising results when using *Virtual LimitLab* as a gaming alcohol-prevention tool. Some technical aspects still need to be improved in order to further refine the prototype, and suggestions for expanding the content of the application have already been made.

## 1. Introduction

Alcohol use is a major risk factor among adolescents for health, social, and legal consequences, including unwanted pregnancy, school failure, and delinquency (1). The 2019 European School Survey Project on Alcohol and Other Drugs (ESPAD) (2) found a higher 30-day prevalence of alcohol use among German adolescent school students compared with the European average (65 vs. 47%). 54% of adolescents in Germany reported having engaged in binge drinking within the preceding 30 days, whereas the European percentage was substantially lower (34%). While 20% of German adolescents in 2019 reported having been intoxicated within the preceding 30 days, the European average was only 13% (2).

Since alcohol consumption typically begins during adolescence and increases with age (3), schools are an important setting for prevention because they are places in which underage peers spend large amounts of time. Reviews that address this age group (4, 5) indicate that interventions (a) should be theory-driven, (b) should address social influences, for example, by helping adolescents to identify social pressures and to resist social influences that encourage alcohol initiation and use (e.g., influences from peers and media), and (c) should support adolescents in building both personal and social skills.

One theory of interventions that aim to prevent alcohol use in adolescence is social inoculation theory (6), which emphasizes strengthening resistance skills to the social pressures faced by adolescents via skills training programs (7). Previous experience with programs that use this theoretical construct have shown positive effects on adolescents' skills in dealing with alcohol (8). Another criterion of effective alcohol prevention in the school setting involves the use of interactive teaching techniques, such as role playing and activities in small groups (4); however, such techniques are time-consuming and expensive to implement at the population level (e.g., they require teachers to undergo specific training).

Virtual reality (VR) is an innovative educational option (9) that allows for creating a virtual learning environment that closely reflects reality. The realistic representation of characters and environments in VR simulations allow users to experience realistic interactions, which crucially stimulates the learning process (10). Positive results of VR as a learning medium have been demonstrated in adolescents' descriptions of VR as a particularly engaging, entertaining, and interesting tool (11). Furthermore, VR simulations technically allow for users to better identify with a simulation and enable environments and interactions to be avatar-specific (12, 13).

The above-described properties of VR can be applied to the field of alcohol prevention because VR enables risky situations to be experienced in a safe environment (14). Currently, only a few VR-based examples of alcohol prevention for adolescents can be found in the scientific literature (15). One example is the Australian alcohol-prevention program Blurred Minds, which was developed as an innovative VR simulation training program for training young people's skills in dealing with peer pressure regarding alcohol (16). Blurred Minds enables VR role playing in which the user is at a birthday party that includes several opportunities to drink alcohol. Initial testing of the simulation revealed that it was appealing to both girls and boys and that it was rated overall with high acceptance and high satisfaction (16). Based on these findings, *VR FestLab*—a prototype with similar objectives—was developed for Danish students in a co-creation process (17, 18) and was later translated and overdubbed for German adolescents with the new name *Virtual LimitLab*. *VR FestLab* was developed based on the taxonomy of the Behavior Change Wheel (19). Accordingly, four behavior change functions are represented in the application, which are presented in an overview in Table 1. While a recent cluster-randomized controlled trial could not demonstrate that the Danish *VR FestLab* simulation had a significant effect on adolescents' alcohol-refusal self-efficacy (20), research on the German *Virtual LimitLab* version has not yet addressed this issue and has instead focused on perceptions of gender in the simulation (21).

**Table 1:** Behavior change function in Virtual LimitLab.

**Behavior change function**	**Description**
Education	During the simulation, the user is confronted with different choices to drink alcohol or to choose a non-alcoholic drink. Blood alcohol concentration is calculated and presented to the user in order to enhance the knowledge regarding the effects of the chosen type of drink on the body.
Training	Different communication options with social feedback are presented in the simulation so that the user can train different communication and behavioral options on how to respond to peer pressure.
Modeling	In the simulation, different role models appear who do not drink alcohol to stimulate learning from these role models how to communicate and behave effectively when choosing not to drink.
Coercion/ incentivization	Negative social consequences for accepting a high number of drinks (e.g., no flirting, vomiting, or blacking out) and incentives (e.g., more behavioral and flirt options and longer party experience) are used to shape realistic expectations toward potential negative outcomes of drinking and positive outcomes of no or moderate alcohol consumption.

Source: own illustration, adapted from Guldager et al. (20).

The objective of the present study was thus to explore German adolescents' content- and technique-specific perceptions of *Virtual LimitLab* in order to better understand the role of user experience in this virtual simulation tool for alcohol prevention. The results of the qualitative analysis should help to further inform the research on VR as a tool for alcohol prevention.

## 2. Material and methods

This exploratory qualitative focus group study aimed to capture adolescents' perceptions of *Virtual LimitLab*. Focus groups were considered as a suitable method because they can be used to effectively collect a number of different positions from different people (22). The study was conducted using the consolidated criteria for reporting qualitative research (COREQ) checklist (23). The completed 32-item COREQ checklist can be found in the Supplementary material.

### 2.1. Virtual LimitLab prototype

**Figure 1:**
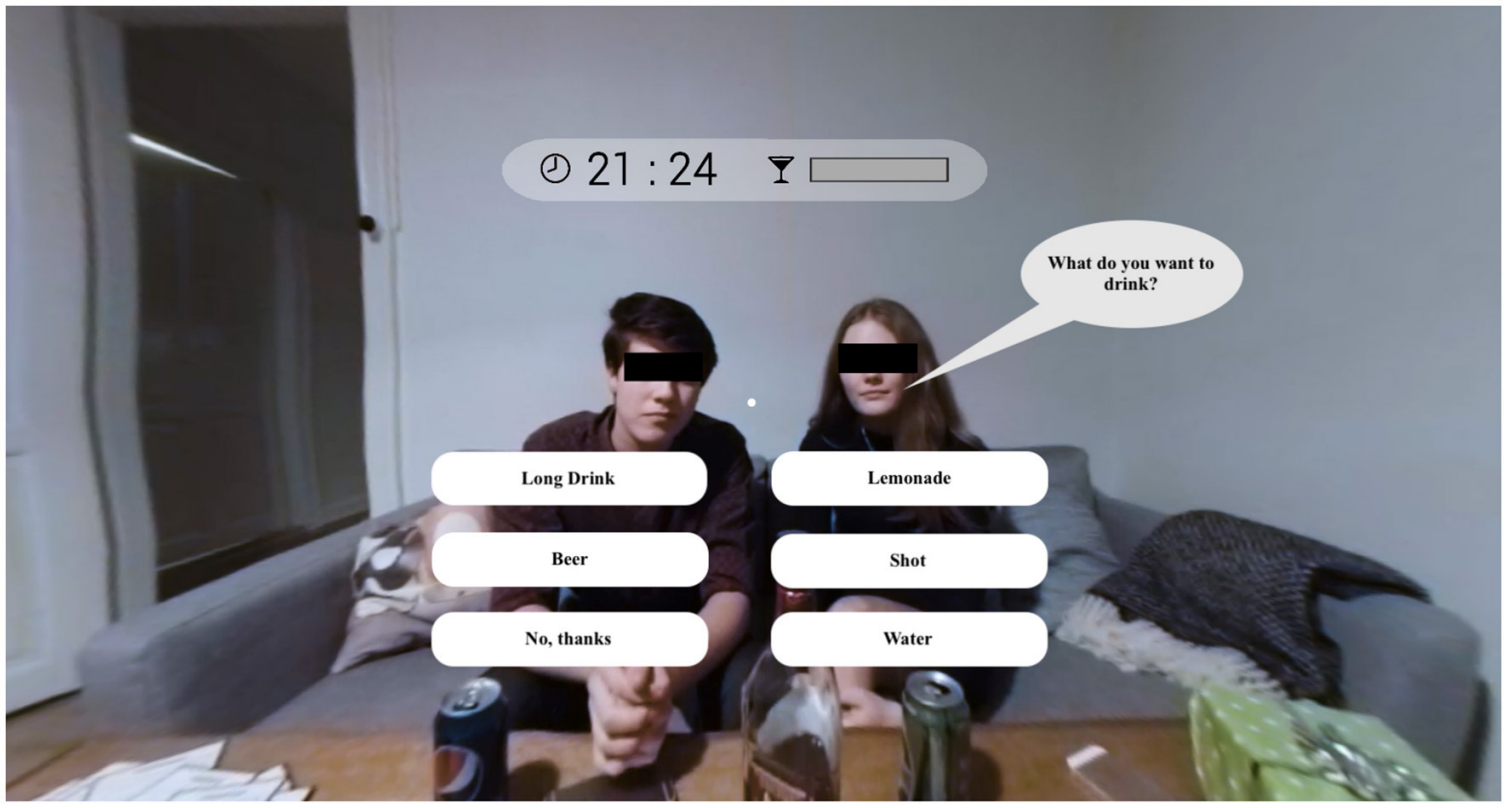
Screenshot of the VR simulation in which the user is asked what they would like to drink text translated to English.

The German VR *Virtual LimitLab* application presents a 360° filmed virtual party simulation consisting of 128 single scenes, which allow for multiple individual pathways based on user decisions during the play. After starting the simulation and selecting a gender, the simulation begins with a pre-party scene in the living room of a fellow school student. The user is accompanied by two other school students, who chat about an upcoming birthday party that all of them intend to join. Afterward, the group attends the party. By using eye movements, the user can navigate through the party and approach different scenes (e.g., beer pong, dancing, or flirting). During the simulation, the user can actively decide to drink alcohol or to choose a non-alcoholic alternative. When choosing to drink alcohol, the blood alcohol concentration (BAC) bar at the top of the screen fills up, which gives the user feedback about their alcohol consumption (Figure 1). The BAC bar was calculated separately for boys and girls using a calculation based on the average weight of a 16-year-old boy or girl, and 1 min was equal to 30 min in the simulation (18). A BAC calculation by Becker and Nielsen (24) was used, which reads as follows:


$$\begin{array}{l} Female\;BAC:\;\frac{Alcohol\;consumed\;in\;grams}{\left(body\;weight\;in\;kg\;x\;60\%\right)-\left(0,15\;x\;hours\;from\;drinking\;start\right)}\\ Male\;BAC:\;\frac{Alcohol\;consumed\;in\;grams}{\left(body\;weight\;in\;kg\;x\;70\%\right)-\left(0,15\;x\;hours\;from\;drinking\;start\right)} \end{array}$$

The script to run the BAC calculation algorithm is executed as soon as the user chose an alcoholic drink. Accordingly, the BAC bar is constantly updated during the game.

**Figure 2:**
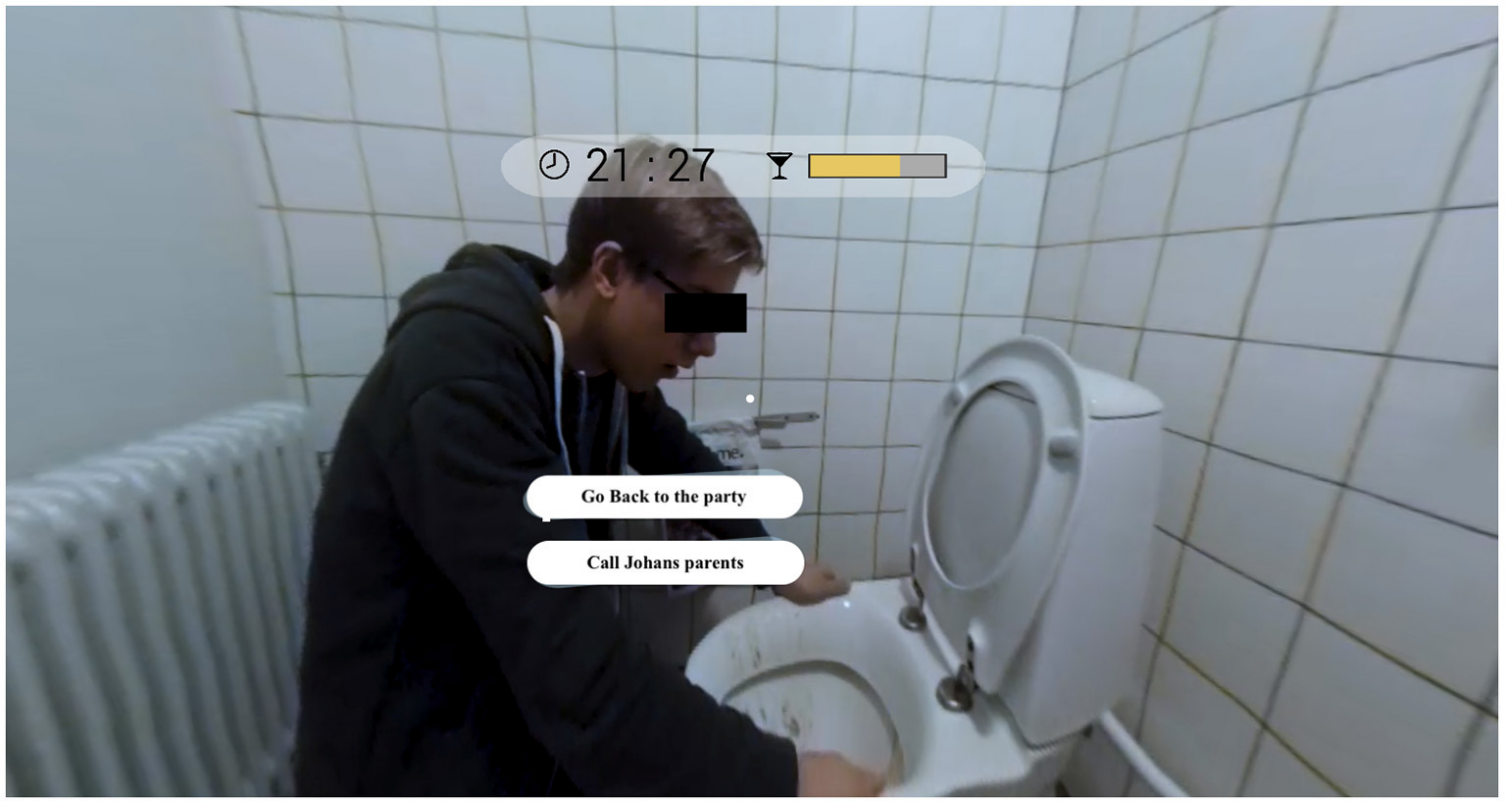
Screenshot of the VR simulation in which the user needs to decide whether to support another party guest text translated to English.

If this BAC bar is filled up completely, the user experiences a blackout, and the simulation ends. In this case, the user is transported to a bedroom and can read a chat on a smartphone display that provides some peer feedback on the user's actions during the party. While some characters encourage the user to consume alcohol, other characters represent role models and refuse to consume alcohol. The aim of the simulation is to increase the user's awareness of how social pressure can influence their own decisions as well as to enable various actions and communication strategies in dealing with alcohol to be trained, as can be seen in a screenshot from a typical scene (Figure 2). Further information on the design of the simulation can be found elsewhere (18).

### 2.2. Participants and ethical considerations

Based on the World Health Organization definition (25) that defines people as adolescents between 10-19 years old, 15-18-year-old adolescents who could understand and speak German were selected, as the Virtual LimitLab application was developed for this age group. Adolescents in this age range were recruited using non-probabilistic convenience sampling techniques. Data saturation was not targeted by using theoretical saturation or stratified sampling as the COVID-19 pandemic severely restricted the recruitment of adolescents (e.g., closing schools and restricting research activities). CP and RH used personal contacts from the Berlin/Brandenburg area. Additionally, youth facilities (*n* = 351) in Berlin were contacted via mass email or telephone. These facilities included youth clubs, cafés, sports clubs, music schools, and local initiatives that cover a wide range of extracurricular activities for adolescents. This diversity of the facilities was deliberately chosen in order to avoid sending invitations to specific subgroups of adolescents. Study information was distributed via email to interested adolescents. This information was the only information that participants had received prior to the study and included the study aims.

Ethical approval was obtained in advance from the ethics committee of the Charité – Universitätsmedizin Berlin (file number: EA2/154/21). Participants were asked to sign an informed consent form prior to data collection. For participants younger than 18, the consent of their legal guardians was obtained in advance. Participants were informed that their study participation was voluntary and that they could withdraw from the study at any time without giving reasons. Furthermore, participants gave their informed consent that the focus groups could be digitally audio-recorded. After participation, the participants received a gift voucher of $36 (€30).

### 2.3. Data collection

Four focus groups with four participating adolescents each were planned and arranged in August 2021 at the Institute of Health and Nursing Science at the Charité – Universitätsmedizin Berlin. The focus groups were implemented by CP and RH—a female and male junior researcher, respectively (each holding a M.Sc. degree in public health), from the institute who had experience in conducting qualitative interviews. CP and RH introduced themselves as researchers interested in further development of digital approaches and explained the scope of the study. Additionally, a student assistant was present and introduced to participants as an assistant who would take down field notes and support the focus groups. Field notes were written after each focus group and included general observations and ideas.

The data collection was divided into two sequential phases. In the first phase, participants in each focus group were informed about the aim and purpose of the study. In this context, the researchers presented their personal background and goals, and the participants had the opportunity to ask questions. Participants were verbally instructed that participation in the study was voluntary and that their participation in the focus group could be terminated at any time without giving reasons. Afterward, the participants tested the VR simulation alone in a room accompanied by a researcher. Navigation through the simulation was explained. Instructions on the goals of the simulation were not given, and the adolescents were instead instructed to explore the simulation independently. Samsung Galaxy S21 devices (Samsung, Suwon, South Korea)—on which the VR *Virtual LimitLab* simulation had been pre-installed—were used to play the simulation. The devices were not able to establish an Internet connection at any time and were inserted into Destek V5 (Thinkline Technology LTD, London, United Kingdom) VR goggles. The application's play time was set to a max. of 20 minutes, which was sufficient to play through the simulation at least once. Depending on which choices were selected and how much alcohol was consumed in the simulation, the simulation differed in playtime. Thus, it was possible for participants to play one or two rounds during the 20-minutes playtime.

While participants were testing the VR simulation, a think-aloud technique was used (26). For this purpose, the participants were encouraged through open-ended questions to talk about the impressions they had while playing the simulation. The think-aloud transcripts were used for a deeper understanding of the focus group results. The thoughts spoken aloud by the users and the remarks of the respective person in the focus group could thus be used to better interpret the participants' statements. The verbal impressions of the adolescents were audio-recorded with a recording device. After completing the testing, a paper-based version of the User Experience Questionnaire—Short (UEQ–S) (27) was handed out to each participant. This quantitative questionnaire was used to triangulate the qualitative results of the focus groups, as statements made by the participants may be influenced by the interview situation. Triangulation from two different data sources (focus group interview and UEQ–S) was used to gain more information. The UEQ–S assessed the user's experience by using a bipolar seven-point scale with pairs of antonymous adjectives. All results were calculated by an Excel sheet (Microsoft Corporation, Redmond, USA), which was provided by the authors of the UEQ–S, on whose website the Excel sheet can be found (28). Values between −0.8 and 0.8 represented a neutral evaluation of the corresponding scale, values >0.8 represented a positive evaluation, and values <-0.8 represented a negative evaluation (28). The UEQ–S showed high internal consistency (Cronbach's alpha) for the sub-scales of pragmatic quality (0.85) and hedonic quality (0.81) (27). Further, a short demographic questionnaire was used to receive more background information about each participant (gender, age, VR, and alcohol drinking experience). In the second phase, all participants met in the same room to discuss their impressions of the VR simulation using the focus group method. The focus groups lasted 30 minutes in total and discussed technical and content-related topics. They were led by a semi-structured interview guide and covered the topics of the perception of the VR simulation, the comparison of the VR simulation with a real party situation, and technical aspects of the VR simulation. These topics were derived from another study (29) in which a Danish VR prototype dealing with alcohol prevention in adolescence was tested. An overview of the semi-structured interview guide can be found in Table 2.

**Table 2:** Guide for the focus group discussion.

1. Opening question:
• How did playing the simulation go for you?
2. Perception of the VR simulation:
• What did you like about the simulation?
• What did you not like about the simulation?
• What would you improve about the simulation?
• When you compare the simulation with other alcohol-prevention programs you know, what comes to mind?
3. Comparison of the VR simulation with a real party situation:
• To what extent does the virtual party portray a real party situation?
• What would you improve in order to make the VR simulation more realistic?
• What do you think about the characters in the VR simulation?
• What do you think about the use of language in the VR simulation?
• Did the characters communicate the same way that you would talk to your friends?
4. Perception of the technical aspects of the VR simulation:
• How would you rate the technical aspects of the VR simulation?
• How did the navigation in the simulation function?
• What would you wish to improve concerning the technical aspects of the simulation?
• Summary:
• Would anyone like to add anything that has not yet been said and that is important?

### 2.4. Data analysis

Both steps of the process (i.e., the think-aloud and the focus groups) were audio-taped, transcribed verbatim, and pseudonymized prior to the analysis. MAXQDA 2022 software (Verbi GmbH, Berlin, Germany) was used for the data organization and analysis. The translation of the German-language quotations into English was carried out by a native English speaker. Both forward and back translation were performed, which ensured that the statements retained their meaning and intention. Methodologically, the thematic analysis developed by Braun and Clarke (30) was selected because the method is not tied to a particular theoretical or epistemological position and therefore retains its flexibility. Braun and Clarke's (30) thematic analysis consists of six steps: (1) becoming familiar with the data, (2) generating initial codes, (3) searching for themes, (4) reviewing the themes, (5) defining and naming the themes, and (6) producing the manuscript. The method is recursive, and newly identified themes and codes can thus be incorporated by returning to previous steps (30).

**Figure 3:**
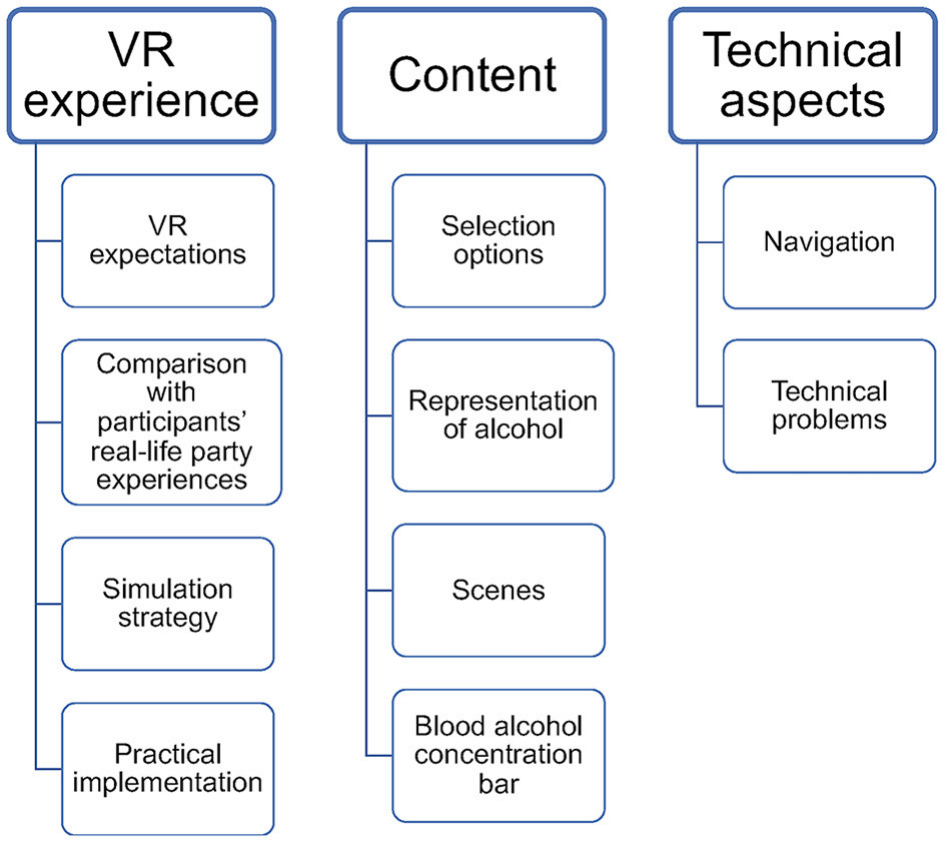
Overview of the thematic map.

First, two researchers (CP and RH) familiarized themselves with the material by matching the transcripts to the audio files and correcting any transcription errors. Then, the transcript was read in its entirety without initial coding. This was followed by initial coding in order to organize the material according to the phenomena of interest. Afterwards, both researchers (CP and RH) searched for themes by checking for internal and external consistency throughout the material and reviewed the themes, defined them, and named them. Subsequently, the themes were applied to all transcripts and the themes and categories were discussed, and refinements were made. Discussions among researchers were used to resolve disagreements. Figure 3 presents a thematic map consisting of the main themes and the inductively developed codes.

## 3. Results

### 3.1. User statistics

A total of 13 participants volunteered to participate during partial lockdown in Germany due to the ongoing COVID-19 pandemic. Three participants declined to participate in the study and did not give any reason for their non-participation. Participants were 16 years old on average. Gender was unevenly distributed [male: *n* = 4 (30%); female: *n* = 9 (70%)]. Ten participants (77%) already had experience with VR. Furthermore, twelve participants (92%) reported having had experience with alcohol. Table 3 displays the participants' demographics.

**Table 3:** Characteristics of the study participants.

	**Total *n* (%)**
**Gender**, ***n*** **(%)**	
Male	4 (30)
Female	9 (70)
Age, mean (SD)	16.08 (1.3)
**Experience with VR**, ***n*** **(%)**	
Yes	10 (77)
No	3 (23)
**Experience with alcohol**, ***n*** **(%)**	
Yes	12 (92)
No	1 (8)

### 3.2. User experience evaluation

**Figure 4:**
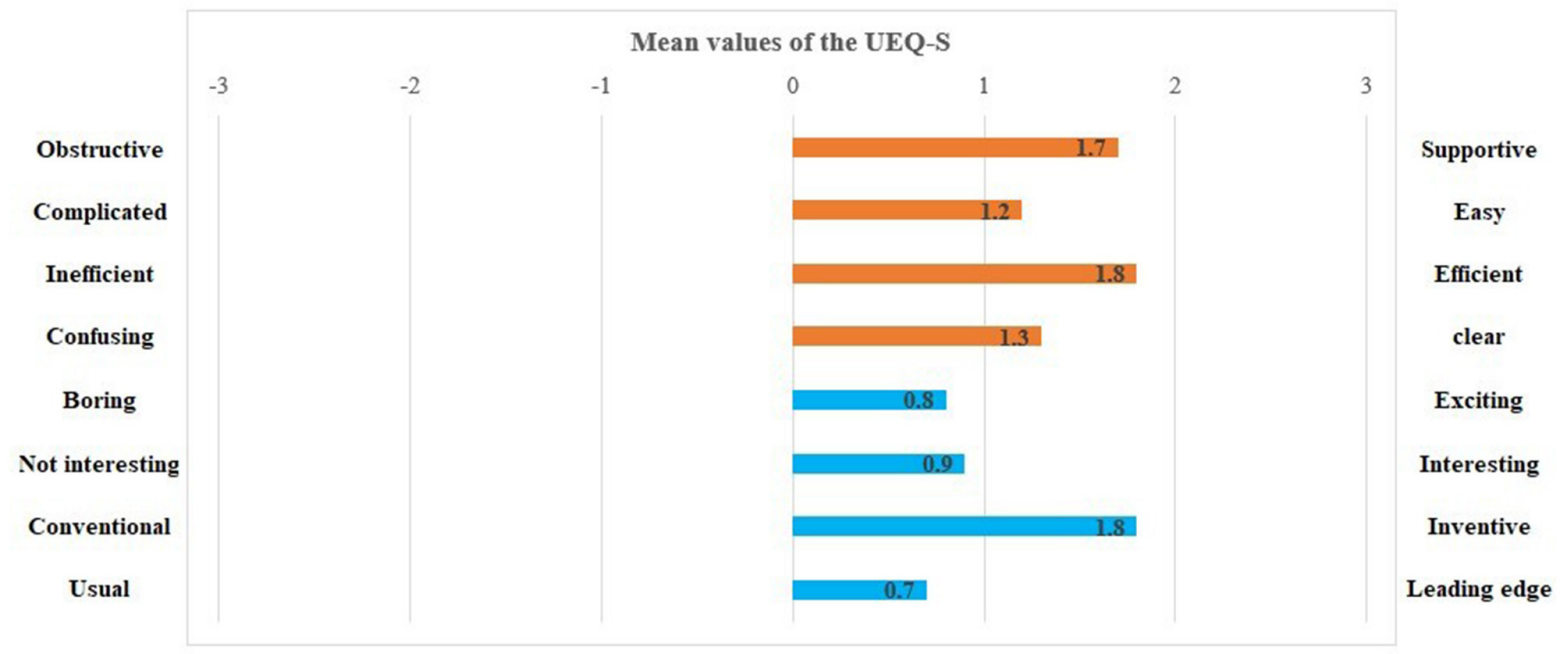
Mean values of the User Experience Questionnaire items (orange: pragmatic quality; blue: hedonic quality).

The UEQ–S showed high means on the pragmatic (mean: 1.05; SD: 0.90) and hedonic (mean: 1.5; SD: 0.90) quality scale. The results of the UEQ–S are depicted in Figure 4. All items except for two (i.e., confusing, and obstructive) showed values >0.8.

### 3.3. Focus group results

#### 3.3.1. VR experience

The first identified theme was *VR experience*, which involved the general expectations that the participants had of VR. The participants discussed their personal experiences with party situations and formulated what they would expect from a VR application in this context. These expectations were also reflected in a discussion of the chosen individual strategies of how to navigate through the simulation. Finally, the implementation of the VR application in the school context was discussed. Four codes were summarized under this theme: VR expectations, comparisons with participants' real-life party experiences, simulation strategy, and practical implementation.

##### VR expectations

The code of VR expectations dealt with the wishes of the participants regarding VR and expressed how the participants viewed VR in the context of party situations and alcohol prevention. Several participants argued that VR should be used to explore and discover the simulation and potential new behaviors. Moreover, some participants stated that VR should present novel and interesting scenes in which the user could immerse themselves. In relation to alcohol prevention, participants positively evaluated the VR simulation's ability to enable them to gain negative experiences without causing them real physical harm (e.g., a hangover or a blackout). This was demonstrated in the following statement:

*B2: [And] you ask, “Okay, if I drink a lot, what will happen? And, like, if I drink a little, what will happen? If I drink too much, what will happen?” So, yeah, that is, like, very helpful in the simulation… [the fact] that you can see… that you can try out [consuming alcohol] without having the experience of a blackout and hangover [in real life] … that you can experience your limit [of alcohol consumption]*.

Another expectation of VR was that the user should be actively involved. Participants felt that the user should be immersed in the simulation and play an active role in the scenes. This was particularly emphasized as a positive element regarding the scenes of playing beer pong, dancing, and drinking alcohol. The participants expected that the VR would enable them to have a sense of space and time in the simulation (*B2: […] So, for me, there was also an immersion… you really are in this place.... and I really felt a little like I was there*.). This was perceived as strengthening the users' bond with the simulation and thus ensured that the users were engaged with the content.

##### Comparison with participants' real-life party experiences

For the code “comparison with participants' real-life party experiences”, the participants compared their personal experiences in party situations and related them to the simulation and to their experiences in the simulation. Most participants perceived the simulated party as a realistic situation:

*A4: I also thought that it [the simulation] was a typical party situation. I've also been to a few birthday parties, and they were actually relatively similar [to the simulation] … there were people dancing, people playing beer pong. At the parties I was at, there were also people on the balcony smoking drugs*.

Even those who had not yet been to a party pointed out that the simulated environment was reminiscent of a film scene (*A1: I've never been to a party, but [the simulation is] a typical party that you know from films, I think. If a situation like that appears in a film, then it looks similar [to the simulation]*.). On the other hand, some real-life party experiences did not align with the simulation experience. For example, one participant described the lack of intoxicating effects of consuming alcohol on the body in the simulation:

*B2: I did not get any feedback on how drunk I actually was. Normally, you have a feeling of how drunk you are, and that was completely missing [in the simulation] … You have this [BAC bar], but you do not have a feeling for it [your actual alcohol consumption]*.

In part, the simulation was also perceived as being somewhat static, which caused some participants to lose interest and led certain actions to result in consequences too quickly (*B3: Well, you could, like, pick up women pretty easily [in the simulation] … you say basically two sentences, and then you could make out. [In real life,] that has not been my personal experience*.).

##### Simulation strategy

The individual participants differed in their chosen strategies to act out the decisions they had made while playing *Virtual LimitLab*. One group chose the strategy of consistently refusing alcohol in their first playthrough. Then, in their second playthrough, they consistently chose to consume alcohol:

*A1: I played two rounds. In the first round, I always said, “No,” and in the second round, I did the exact opposite. [I noticed that] the first round was significantly longer … it was as if you were having a blackout in the second round, and then you suddenly woke up in bed*.

The participants explained that the different simulation strategies were reflected in their choice to interact with diverse characters with different behavioral options. Then again, some participants did not pursue a strategy and allowed themselves to drift spontaneously through the simulation. These participants paid less attention to the BAC bar. In general, the interviews did not indicate that the participants' chosen simulation strategy was oriented toward real-life behavior. Instead, different strategies were tried out in relation to low, moderate, and high levels of alcohol consumption:

*D3: I played two rounds and then started a third, and at first, I drank a little bit of alcohol… in the second round, [I drank] a lot, and in the third round, [I didn't drink] at all*.

##### Practical implementation

Finally, the participants commented on how *Virtual LimitLab* could be implemented in practice. Some participants regarded *Virtual LimitLab* as a decision-making aid for exploring how adolescents feel in party situations. In principle, the participants were in favor of using *Virtual LimitLab* in schools; however, one participant favored playing alone and in a protected space since teachers and other adolescents would be able to receive personal information if they observed the user (e.g., during a scene in which the user is asked whether they have ever had sex). Participants felt that a combination of traditional prevention strategies (i.e., providing information on the consequences of harmful alcohol consumption) and VR simulation techniques would be beneficial. According to the adolescents, the gaming approach of *Virtual LimitLab* can be used to arouse interest in the topic, which could then be enhanced by health education on alcohol consumption (*C2: Then, you don't have either only the normal prevention [strategy] or only the virtual-reality version, but you can combine both, and I think that makes sense*.).

#### 3.3.2. Content

The second identified theme was the *content* that emerged when the participants were asked about positive and negative aspects of the simulation and about their corresponding suggestions for improvement. Four different codes developed and were discussed in terms of content: selection options, the representation of alcohol, scenes, and the blood alcohol concentration bar.

##### Selection options

Adolescents' perceptions of the selection options in the VR simulation were central to the theme of *content* because these perceptions involved the users' interaction in the simulation.

Participants agreed that *Virtual LimitLab* is entertaining and exciting due to the variety of available choices (*A4: Yes, I thought it was cool that you had so many options of what to do first [in the simulation], like [playing] beer pong*.). Furthermore, they described *Virtual LimitLab* as a simulation in which behaviors could be practiced that could not necessarily be practiced in real life:

*A1: I think it's good that there are choices [in the simulation] because… it's a simulation, and you should also try out new things that you wouldn't do in everyday life to see what it [the new behavior] is like*.

Participants explicitly addressed the possibility of selecting options to interact with the characters at the party since these options were important to the participants in their discussions about VR. Nevertheless, there was no consensus regarding the optimal number of selection options. While some participants felt that the selection options were sufficient and very detailed, other participants stated that the selection options lacked variety and gave the user limited choices. Some options in the simulation were perceived by some participants as not appropriate and hindered their ability to identify with the simulation character (*D3: Sometimes, there were only two options, and if you chose one, you were immediately taken out of the situation again, and I didn't like that very much... I'd rather have more options*.).

Hence, some of the options in the simulation were perceived as unrealistic. At the same time, participants criticized the fact that if some options were chosen, the scene ended quite quickly, and the user could no longer experience the situation. However, other participants described the variety of choices as quite diverse and felt that this diversity made the simulation very interesting (*B3: I would actually say...the many choices [for responding was a positive thing]. There were really a lot of options that you could choose [in the simulation] … that you could interact with*.). The participants' statements indicated that the variety of choices aroused interest in playing and re-playing the simulation (*B4: Yeah, it [the simulation] was over pretty quickly. There were, like, a lot of options at the end, and you thought, “Ah, yes, I could do something different [next time].”*).

##### Representation of alcohol

The topic “representation of alcohol” was not introduced by the moderator. On the one hand, the participants praised the simulation for not conveying a one-sided, negative view of alcohol consumption. Previous experience with alcohol prevention at schools was found to be negative because such approaches often condemn the consumption of alcohol and its consequences:

*A1: I actually think it's good that it is [the consequences of drinking alcohol] not always portrayed as extremely as in all the educational films that you have to watch at school, [in which] everyone is drunk as a skunk and basically ends up on the street or something, and their life is over now because they got drunk once or something. I think it's good that it is [the consequences of drinking alcohol] portrayed a bit more mildly [in the simulation]*.

On the other hand, participants disagreed regarding the depicted consequences of alcohol consumption in the simulation, with some participants stating that the negative consequences of drinking alcohol should be better portrayed in the simulation. Participants also disagreed on whether these consequences should be particularly shocking and extreme or whether they should be milder. In general, the participants agreed that the effects of consuming alcohol in the simulation differed from their own experiences with alcohol. They mentioned that the simulation user does not necessarily experience the effects of consuming alcohol that would normally be experienced in real life as physical or mental symptoms. Suggestions were made to improve the simulation in this respect, for example, by introducing blurred vision or incorporating more serious consequences. In this context, mental blackouts were frequently mentioned as a possible serious consequence of alcohol consumption that should occur in the simulation after excessive alcohol consumption and that should be represented by embarrassing party pictures (e.g., photos of the user throwing up or performing embarrassing acts at the party) that are shown to the user in the aftermath in the simulation:

*A2: [...] but maybe, somehow, there could be a sequence where you see pictures of the party [from] when [the party] at some point just completely escalated and where you think, “Oh, what did I do?” … where there could be a surprise effect at the end*.

In *Virtual LimitLab*, this mental blackout is represented by ending the simulation as soon as too much alcohol has been consumed and by having the user wake up in a bedroom. However, the participants' statements indicate that not all users interpreted this sequence as a blackout. Instead, the scene was viewed as teleportation to a bed where the user wakes up:

*B2: I think you really… you, like, really have to experience it [a blackout in the simulation]. Being teleported to a room is not a blackout. If you can really have this experience [of blacking out in the simulation], then it would also help you a lot to have a better relationship with alcohol*.

As a general weakness of VR, some participants mentioned the fact that VR is merely virtual and that the effects and actions that occur within the simulation do not perfectly correspond to those in real life. On the other hand, the following quotation also demonstrates that the simulated environment was perceived positively and that only the discrepancies between the simulation and reality were perceived unsatisfactorily, which remains a difficult issue for VR applications to solve:

*B4: Yes, I think the conversations were great. When you dance around at a party for a while [in real life] … you can't really do that [dancing] virtually, when you're just, like, standing around, when you haven't really drunk anything… it is [the experience in the simulation] just different*.

##### Scenes

Some participants would have liked to see more scenes in addition to the house party, such as a party in a park (*A4: Or you could have two different versions of a party. You could have a party in a house [and] a party in a park because [in the party in the park version,] you could also be approached or harassed by strangers*.). Other participants explained that they would have liked to experience how to get home drunk after the party (*D1: I would find it cool to see how to get home when you're drunk*.). Both quotes illustrate that the participants had different ideas about parties in their age group. Whereas the first quote addresses possible social consequences of alcohol consumption (e.g., harassment by strangers), the second quote suggests that the scenes in the simulation focus more on the altered sensory perceptions caused by excessive alcohol consumption *via* a simulated experience of alcohol intoxication (e.g., walking home drunk).

The perception of the language used in the simulation was also evaluated in different ways. On the one hand, the characters and their language use were perceived as realistic because they used appropriate adolescent language that did not sound staged:

*B3: I thought it was really good that the characters weren't like in other school videos, where there are some young people who frantically try to use some slang. I don't like that at all, and I think that's why I got so involved in the simulation*.

On the other hand, the voices of the characters were perceived as somewhat artificial, which made the characters seem less realistic:

*A1: These voices are the typical German overdubbing voices. The most famous [overdubbing] actors were packed into a room and then recorded. […] It was hard to connect the voices to the people themselves because they just... spoke more like an overdubbing voice [actor] than like a person who exists in real life*.

##### Blood alcohol concentration bar

The participants had differing perceptions of the blood alcohol concentration (BAC) bar, which was displayed at the top of the screen as a yellow bar next to the time in the simulation (Figure 1). Some of the participants correctly interpreted the BAC bar as the representation of their current blood-alcohol concentration. Those who correctly interpreted the BAC bar modified their behavior in the simulation based on the bar's status (*A3: Well, first, I played beer pong with the guy there, then I saw [based on the BAC bar] that I could drink some more, then I drank something else […]*.). These participants rated the BAC bar as helpful because they were better able to assess their drinking behavior (*A3: I thought it was nice to have the bar, but in real life, you don't have that bar and are more sensitive [to alcohol] than you think*.).

One participant was critical of the BAC bar because it could trigger greater alcohol consumption due to the perceived pressure to fill up the bar. The BAC bar was also criticized for being an overly scientific construct. The normative recommendation that the user's alcohol consumption be represented by the BAC bar was thus partly rejected.

*B2: I generally don't like it [the BAC bar] because it says so scientifically how much you still have to drink and how much you are still allowed to drink. The bar of how much more you can drink doesn't exist in real life*.

The other group of participants did not interpret the BAC bar correctly and assumed that it had other functions (see Figure 1). While some thought that the yellow bar represented the seconds in the simulation, others did not notice the bar at all in the simulation:

*A2: I only looked at the time at the top [of the screen], and I somehow thought the yellow bar represented the seconds. I was super unsure [about it] and thought it must mean something. I just, like, drank and drank the whole time, but I didn't pay any attention to it*.

#### 3.3.3. Technical aspects

The third theme that emerged involved the *technical aspects* of the simulation when the participants were asked about their opinions of the technique employed by the simulation. The theme of *technical aspects* was divided into the two codes of navigation and technical problems.

##### Navigation

Regarding the technical aspects of *Virtual LimitLab*, most expressed satisfaction with the simulation. The ease of using the navigation was highlighted as a positive aspect (*B4: Well, I thought the technical aspects of VR were rather simple because you, like, can't do much with only a pair of glasses. So, I thought it [the simulation] was well implemented considering the circumstances […]*.). In this context, the participants discussed the possibility of adding further controls to the simulation. Basically, the participants found that using eye movements to control the simulation was sufficient for *Virtual LimitLab*. Several participants wanted their own movement to be displayed in the simulation because they felt that it would have made the simulation more immersive in interactive scenes. One participant suggested technical improvements that would help to even better immerse users in the simulation (*A3: I would have liked to be able to run around … At some point, I was dancing, but I couldn't really dance [in the simulation] like I would in reality […]*.). Apparently, it was important to the participants that the VR user be able to identify with the actions in the simulation and to perform these actions both virtually and in reality. When actions (e.g., running or dancing) in the simulation did not match real-life movements, it was not possible for participants to feel fully immersed in the simulation.

##### Technical problems

While some participants emphasized the simple and user-friendly design of the simulation, others addressed technical problems, as described in the following quote:

*C2: I would make the selection options less transparent so that you can see them a bit better. Sometimes, I saw things double, which could be due to how crooked I had my glasses on or to the fact that I wasn't wearing contact lenses or glasses at the time. And I would have liked to have a button in the cell phone scenario where I could get back to the other chats, where I could look at [the return button]… that it is a bit more clear where I have to look in order to get back… because I first had to ask myself, “Hey, how do I get away from it [this screen]?”*

Some participants also reported that they could not recognize faces correctly in the VR simulation or that texts were not readable. While some of these statements may have been due to the incorrect handling of the VR glasses, some participants criticized the short duration in which the texts in the simulation were displayed, which left the user with too little time to read them carefully. The participants suggested adding a function that would allow the user to jump forward or backward a few scenes and to navigate at their own pace (*B4: […] but also, like, sometimes, [it would be great if] you could go back a few seconds if you didn't understand something, or that you could skip certain content […]*.).

Participants also described problems that were caused by malfunctions, which left them stuck in the simulation. Several participants also found that the end scene had an overly long play time that literally trapped them in the simulation for about 60 seconds until they had played through the simulation and woken up in the bed scene.

## 4. Discussion

This study provided valuable insights into adolescents' perceptions of a VR-based alcohol prevention tool, and three main themes emerged: *VR experience, content*, and *technical aspects*. To summarize the results of the focus groups, we found that adolescents evaluated the prototype positively, and *Virtual LimitLab* was frequently described as innovative and interesting. This trend was also reflected in the quantitative UEQ–S data, which yielded the highest values for the adjectives “interesting” and “leading edge,” both of which corresponded to the qualitative statements in the focus groups. The ease of use was also qualitatively highlighted in the theme of *technical aspects* and was seen by a positive rating of the corresponding UEQ–S item.

An overall positive evaluation of a VR simulation designed for alcohol prevention has also been demonstrated by Guldager et al. (29), who tested the Danish *VR LimitLab* application with adolescents. Since *Virtual LimitLab* is the German version of the same simulation, we can conclude that the application is well accepted by young people in “wet” drinking cultures, such as Denmark and Germany. Positive perception of VR by adolescents has also been demonstrated in other areas of application (31–33). For example, Farič et al. (31) developed a VR application for physical activity and reported that adolescents evaluated it positively and saw great potential for using VR in that context. In particular, the playful and low-threshold approach of VR was welcomed by adolescents.

Our results are comparable to those of Farič et al. (31) and indicate that VR could be successfully used in the field of alcohol prevention. VR is interesting to adolescents and represents an innovative medium for them. Current preventive measures—which often take the form of posters, workshops, and lectures and are based on the assumption that adolescents choose their behavior rationally—have shown only limited effects (34) and acceptance in the target group (35). VR could overcome this barrier by addressing preventive actions at the target group through gamification in an appealing and engaging way. The ability of VR to enable adolescents to try out actions and behaviors and to gain new experiences through immersion is a new way of getting them excited about prevention issues and addressing their emotions rather than addressing only their rational decision-making skills.

However, the adolescents in our study suggested that *Virtual LimitLab* could be combined with other prevention activities. To that end, the adolescents suggested that *Virtual LimitLab* could be used as a thought-provoking stimulus for alcohol prevention that is then further elaborated in group discussions and with other evidence-based alcohol-education elements in more comprehensive school-based programs. Research is needed that identifies preventive intervention components that could be combined with virtual refusal-skills simulations in order to maximize the effects of alcohol-prevention programs. Alternative evaluation methods (e.g., sequential multiple-assignment randomized trials, factorial designs) (36) that can be used to address this issue already exist.

On the other hand, alcohol prevention for adolescents does not necessarily need to take place in schools. Farič et al. (37) concluded that the use of VR is welcomed by adolescents because it is independent of time and space. Home-based VR could help to reduce cultural and social barriers that might exist at school due to peer pressure (37). Using digital alcohol-prevention applications at home is conceivable because the equipment (i.e., VR goggles) is affordable nowadays. Prices range from $9.00 (€8.01) to $39.95 (€35.56). If *Virtual LimitLab* is offered as a free app, adolescents could deal with the topic independently and at their own pace. However, alcohol-prevention apps that are used at home may not reach their full preventive potential because the social environment could interfere with the apps' messages and intentions. The school setting could thus ensure that the VR program is properly used via teacher support.

A general challenge for VR is to provide users with a realistic immersion (38). Virtual environments (e.g., a party setting) must be perceived as realistic so that the user can identify with the space into which they are introduced. According to our results, *Virtual LimitLab* was able to successfully immerse adolescents into the simulation space because the adolescents perceived the simulation as realistic. Even adolescents who had limited or no real-life party experience were convinced that a party in their age group would be similar to the one presented in the simulation. The participants' perceived realism could also be discussed in the context of health behavior change models, such as the Protection Motivation Theory (39). Research on fear appeals in substance use prevention highlighting the negative consequences of risk behaviors do not come to conclusive results, and these strategies are found to be insufficient to result in sustained behavior change (40). Our study revealed that participants perceived the VR application and the displayed behavior and consequences of alcohol use as realistic, but we could not collect information on whether the VR experience resulted in a perceived severity of a health threat or in a perceived personal vulnerability by alcohol misuse. It can also be critically discussed whether the participants' assessment of coping options is sufficiently triggered to induce a change in behavior. These open questions should be addressed in further studies.

Moore et al. (41) performed semi-structured interviews on user experience in their development of a VR-based advanced life-support-training platform and found that their respondents felt that realism is crucial to user engagement. Thus, the positive results of the user-experience rating in our own study could be explained because the adolescents perceived *Virtual LimitLab* as realistic. User engagement should be centrally addressed in the further development of *Virtual LimitLab* because it determines the success or failure of VR applications in terms of their implementation in practice (42).

The perceived immersion of the adolescents in our study might further be explained by the participatory development process of *Virtual LimitLab*. The simulation was developed together with adolescents in a co-creation approach that enabled a close exchange of ideas between researchers, technical developers, and the user group of the intervention (18). Co-creation represents a promising approach for developing effective prevention interventions that are accepted by the target group, especially in VR. However, such a co-creation process cannot guarantee that there are no adolescents who are critical of the intervention. For example, the representation of alcohol in the simulation was addressed as an opportunity for improvement by some adolescent users since it was not considered to be frightening enough. On the other hand, adolescents welcomed the absence of appeals to fear concerning alcohol in the simulation. This dichotomy must be addressed in the further development of *Virtual LimitLab*. In principle, it is feasible to represent serious alcohol-related risks (e.g., blurred vision) in the simulation, but this could lead to a diminished simulation experience because cyber sickness could become more likely.

Another aspect to be considered in future versions of VR simulations is the role of gender in the flirting options because some participants raised the concern that the flirting experience was not optimally portrayed. Prediger et al. (21) indicate that aspects of flirting, sexual harassment, and gender issues could well be incorporated into gender-sensitive VR simulations addressing alcohol consumption among adolescents. Due to the relatively homogenous group of respondents with respect to age and prior alcohol and VR experiences, we could not identify any hints on how age or prior experiences might have influenced the perception of *Virtual LimitLab*. Other research indicates that younger adolescents and those without prior alcohol experience might be more receptive toward virtual alcohol prevention applications (20).

In their qualitative study, Guldager et al. (29) described a concern of adolescent users that was also mentioned in our study. Those who rejected alcohol consumption in the Danish simulation perceived it as more tedious and monotonous than not consuming alcohol. These results can be used to future develop *Virtual LimitLab*. Non-consumption options should be at least equally attractive and entertaining in the simulation. Since the preventive aim is to discourage alcohol consumption, special care must be taken to ensure that the gamification approach does not lead to unintended effects (43). Although the effectiveness of *Virtual LimitLab* on drinking outcomes has not yet been studied, the results of a cluster RCT (20) conducted on the effectiveness of the Danish *VR FestLab* simulation on alcohol-refusal self-efficacy did not show any counterproductive or unintended effects. This result indicates that although adolescents consider the alcohol choices in the simulation to be fun, this does not necessarily mean that alcohol consumption becomes more attractive to the target group while playing the simulation. Based on the evaluation results of the Danish application *VR FestLab* (20), this qualitative study of *Virtual LimitLab* helps to further improve the simulation and subsequently undergo an evaluation to examine the effects of an optimized tool.

Technical issues are especially important in VR applications as they can hinder the desired immersion and can hamper the VR experience. Special attention in the future development of *Virtual LimitLab* should be paid to the technical aspects of the simulation. Our study revealed that adolescents found these technical aspects to be sufficient and repeatedly emphasized the simplicity and clarity of the simulation. On the other hand, recommendations were made to further enhance the immersive experience of the simulation. Since *Virtual LimitLab* relies on the interaction of the user with the environment, virtual body movements could be added that mimic the user's real movements. When creating or modifying active simulation components (e.g., playing beer pong), real body movement that is integrated into the simulation could be considered as a means of enriching user experience. By matching real and simulated body movement, a higher level of immersion could be created that might also enhance the simulation's preventive effects.

## 5. Limitations

This study has several limitations that must be considered when interpreting its results. First, the study population was small, with only 13 participants. The quantitative results of the UEQ–S must also be interpreted with caution under this limitation. Due to the small sample size, the results of the UEQ–S are biased, not representative, and were only used to triangulate the qualitative data. Therefore, they can only be interpreted as an indicator for the study participants. Although the size of the study population is not critical in qualitative studies (44), data saturation was not aimed for and can be seen as a limitation. Unfortunately, the recruitment of adolescents aged 15–18 years was considerably hampered due the COVID-19 pandemic measures that were in place during recruitment, with well-suited recruitment sites—such as youth clubs—having been closed due to contact restrictions. Snowball recruitment was thus particularly successful and yielded several participants; however, the disadvantage of this recruitment strategy is that it is prone to self-selection bias. Therefore, it was difficult to identify extreme cases that might have enriched the data. A recruitment bias might have been present because participants were primarily high-school students, and participants from middle school were not represented in the sample. Due to reduced capacities in terms of time and personnel, no repeat interviews were conducted. Such repeat interviews might have improved the data quality. In addition, the transcripts were not sent back to the participants for comments, and the participants therefore could not provide any further feedback. These limitations were recorded in the COREQ checklist (Supplementary Table 1).

Second, the role of social desirability cannot be ruled out because the participants were informed that the study involved a VR application for alcohol prevention, and the discussions may have been biased toward prevention benefits. However, the guided focus groups included a variety of topics, and any existing role that social desirability may have played may not have been maintained throughout the entire discussion.

Third, due to the limit of four adolescents per focus group, certain adolescent who were highly vocal and convincing in their opinions may have influenced the discussion. To prevent this, the moderator made an effort to actively include each person in the discussion. Additionally, it is not possible to rule out whether the participants who took part in the study had existing positive feelings toward VR or were highly experienced with it. This limitation is reflected in the observation that only 3 of 13 participants stated that they had not had any experience with VR. On the other hand, participants who were experienced with VR might have had higher expectations for the new VR simulation, which could have resulted in a more critical perspective toward the prototype.

Fourth, results must be seen in the context in which they were collected. While user experiences of the simulation were overall positive in Denmark and Germany—countries with “wet” drinking cultures—there may also be adolescents with differing drinking cultures (micro-level) within these national drinking cultures (macro-level) (45) for whom such a simulation might be less appealing. In addition, adolescents with little affinity for technology and aversion to VR, who may view technological innovations critically may be less positive than the adolescents who participated in the study. Therefore, more research with diverse adolescents is needed to determine for which target groups VR simulations work best and to develop approaches for those most at risk.

## 6. Conclusions

*Virtual LimitLab* is the first VR-based alcohol-prevention simulation in Germany that aims to promote alcohol-refusal self-efficacy among adolescents. The present study on user experiences with the German prototype revealed that the approach is welcomed and accepted by German adolescents. Participants highlighted the simulation's good usability and technical aspects. User experience with *Virtual LimitLab* was positively assessed, but more research is needed in order to determine the simulation's effects on alcohol choices and behavioral outcomes.

## Data availability statement

The datasets presented in this article are not readily available because of data protection. Requests to access the datasets should be directed to RH, robert.hrynyschyn@charite.de.

## Ethics statement

The studies involving human participants were reviewed and approved by Ethics Committee of the Charité – Universitätsmedizin Berlin (file number: EA2/154/21). Written informed consent to participate in this study was provided by the participants' legal guardian/next of kin.

## Author contributions

Conceptualization: RH, CP, PL, GM, and CS. Methodology, formal analysis, recruitment, and focus group organization: RH and CP. Writing and preparation of the original draft and visualization: RH. Review and editing: RH, CP, SH, PL, GM, and CS. Supervision, project administration and funding acquisition: CS. All authors have read and agreed to the published version of the manuscript.

## Supplementary material

Supplementary Table 1 Consolidated Criteria for Reporting Qualitative Studies (COREQ): Completed 32-item checklist.
