# Corrigendum: Adolescents' perceptions and user experiences with a virtual reality-based alcohol prevention tool in Germany: A focus group study

**DOI:** 10.3389/fpubh.2024.1447472

**Published:** 2024-06-26

**Authors:** Robert Hrynyschyn, Christina Prediger, Patricia Lyk, Gunver Majgaard, Stefanie Maria Helmer, Christiane Stock

**Affiliations:** ^1^Charité – Universitätsmedizin Berlin, corporate member of Freie Universität Berlin and Humboldt Universität zu Berlin, Institute of Health and Nursing Science, Berlin, Germany; ^2^Leibniz Science Campus Digital Public Health, Bremen, Germany; ^3^University of Southern Denmark, Game Development and Learning Technology, The Maersk Mc-Kinney Moller Institute, Odense, Denmark; ^4^University of Bremen, Human and Health Sciences, Bremen, Germany; ^5^University of Southern Denmark, Unit for Health Promotion Research, Esbjerg, Denmark

**Keywords:** virtual reality, user experience, alcohol prevention, adolescents, focus group, qualitative research

In the published article, there was an error in the funding statement. We did not state that the development of the virtual application that was tested in the study (Virtual LimitLab) was co-funded by a donation from TrygFonden, who funded the development of the original Danish version VR FestLab. The funding statement was originally written as follows: The research project was funded by Stiftung Charité (file number WIS_PRO_619). The study sponsor and funders had no role in the study design, data collection, data management, data analysis, data interpretation, or report writing or in the decision to submit the report for publication.

The correct Funding statement appears below.

