# Supplementary material for: Adolescents' perceptions and user experiences with a virtual reality-based alcohol prevention tool in Germany: A focus group study
Source: Front Public Health. 2023 Mar 10;11:1054015. doi: 10.3389/fpubh.2023.1054015 (PMC10038231; doi:10.3389/fpubh.2023.1054015)
Supplement: Supplementary Table 1 — Consolidated Criteria for Reporting Qualitative Studies (COREQ): Completed 32-item checklist. [file Data_Sheet_1.pdf]

Supplementary Material

1 Supplementary Data

Supplementary Table 1. The Consolidated Criteria for Reporting Qualitative Studies (COREQ): completed 32-item checklist

Topic	No. Item	Guide Questions/description	Reported on Page
**Domain 1: Research team and reflexibility**			
Personal characteristics			
Interviewer/facilitator	1	Which author/s conducted the interview or focus group?	4
Credentials	2	What were the researcher's credentials? e.g. PhD, MD	4
Occupation	3	What was their occupation at the time of the study?	4
Gender	4	Was the researcher male or female?	4
Experience and training	5	What experience or training did the researcher have?	4
Relationship with participants			
Relationship established	6	Was a relationship established prior to study commencement?	4
Participant knowledge of the interviewer	7	What did the participants know about the researcher? e.g. personal goals, reasons for doing the research	4
Interviewer characteristics	8	What characteristics were reported about the interviewer/facilitator? e.g. Bias, assumptions, reasons and interests in the research topic	4

Domain 2: study design

Theoretical framework			
Methodological orientation and Theory	9	What methodological orientation was stated to underpin the study? e.g. grounded theory, discourse analysis, ethnography, phenomenology, content analysis	2
Participant selection			
Sampling	10	How were participants selected? e.g. purposive, convenience, consecutive, snowball	3
Method of approach	11	How were participants approached? e.g. face-to-face, telephone, mail, email	3
Sample size	12	How many participants were in the study?	6
Non-participation	13	How many people refused to participate or dropped out? Reasons?	6
Setting			
Setting of data collection	14	Where was the data collected? e.g. home, clinic, workplace	3
Presence of non-participants	15	Was anyone else present besides the participants and researchers?	4
Description of sample	16	What are the important characteristics of the sample? e.g. demographic data, date	Table 3
Data collection			
Interview guide	17	Were questions, prompts, guides provided by the authors? Was it pilot tested?	Table 2
Repeat interviews	18	Were repeat interviews carried out? If yes, how many?	N/A
Audio/visual recording	19	Did the research use audio or visual recording to collect the data?	5
Field notes	20	Were field notes made during and/or after the interview or focus group?	4
Duration	21	What was the duration of the interviews or focus group?	5
Data saturation	22	Was data saturation discussed?	12
Transcripts returned	23	Were transcripts returned to participants for comment and/or correction?	N/A

Domain 3: analysis and findings

Data analysis			
Number of data coders	24	How many data coders coded the data?	5
Description of the coding tree	25	Did authors provide a description of the coding tree?	Figure 3
Derivation of themes	26	Were themes identified in advance or derived from the data?	5
Software	27	What software, if applicable, was used to manage the data?	5
Participant checking	28	Did participants provide feedback on the findings?	N/A
Reporting			
Quotations presented	29	Were participant quotations presented to illustrate the themes / findings? Was each quotation identified? e.g. participant number	6-10
Data and findings consistent	30	Was there consistency between the data presented and the findings?	6-10
Clarity of major themes	31	Were major themes clearly presented in the findings?	6-10
Clarity of minor themes	32	Is there a description of diverse cases or discussion of minor themes?	6-10

(Table developed from: Tong, Allison, Peter Sainsbury, and Jonathan Craig. "Consolidated criteria for reporting qualitative research (COREQ): a 32-item checklist for interviews and focus groups." International journal for quality in health care 19.6 (2007): 349-357.)
